# Investigation of Biological Activities of Wild Bitter Melon (*Momordica charantia* Linn. Var. Abbreviata Ser.)

**DOI:** 10.3390/biom9060211

**Published:** 2019-05-30

**Authors:** Thi My Hanh Pham, Dai-Hung Ngo, Dai-Nghiep Ngo, Thanh Sang Vo

**Affiliations:** 1 Vo Van Kiet Senior High School, Ho Chi Minh City 700000, Vietnam; hanhpham3006@gmail.com; 2 Faculty of Natural Sciences, Thu Dau Mot University, Thu Dau Mot City 820000, Binh Duong province, Vietnam; 3 Faculty of Biology and Biotechnology, University of Science, Vietnam National University, Ho Chi Minh City 700000, Vietnam; ndnghiep@hcmus.edu.vn; 4 NTT Hi-Tech Institute, Nguyen Tat Thanh University, Ho Chi Minh City 700000, Vietnam

**Keywords:** *Momordica charantia*, wild bitter melon, DPPH, α-amylase, nitric oxide

## Abstract

Wild bitter melon (*Momordica charantia* L. var. Abbreviata Ser.) is a wild edible variety of *M. charantia*, often used in folk medicine. In this study, the biological activities of its extract and fractions were investigated in vitro. It was found that ethyl acetate (EA) fraction exhibited high 1,1-diphenyl-2-picryl-hydrazyl (DPPH) scavenging activity with a half maximal inhibitory concentration (IC_50_) value of 0.43 ± 0.04 mg/mL, while the chloroform (CF), EA, and n-butanol (Bu) fractions had strong 2,2-azinobis-3-ethyl benzothiazoline-6-sulfonic acid (ABTS)^+^ scavenging ability with IC_50_ values of 0.36 ± 0.04 mg/mL, 0.35 ± 0.02 mg/mL, and 0.35 ± 0.05 mg/mL, respectively. Moreover, the EA and Bu fractions exhibited the highest protective effect against H_2_O_2_-induced DNA damage in a concentration-dependent manner. Furthermore, the EA fraction was effective in the inhibition of enzyme α-amylase activity with an IC_50_ value of 0.27 ± 0.029 mg/mL. Finally, it was observed that the production of nitric oxide (NO), a pro-inflammatory mediator, was significantly reduced from LPS-stimulated murine macrophage RAW 264.7 cells by the ethanol extract (ET) and the EA fraction. Therefore, wild bitter melon could be considered as a promising biomaterial for the development of pharmaceutical products.

## 1. Introduction

Natural products are well known for their unique structural diversity, which can be used for the development of novel pharmaceutical products [1,2,3]. While 10% of the world’s biodiversity has been evaluated for potential biological activity, the remaining 90% is still awaiting assessment [4]. Notably, plants have been documented for their medicinal uses for thousands of years. It is estimated that plant-based traditional medicines are still widely used for primary health care, and their original ethnopharmacological properties are related to 80% of drug discovery [5,6]. The effectiveness of traditional medicines has inspired further studies into the use of medicinal plants for the discovery of potential drugs.

*Momordica charantia*, known as bitter melon, belongs to the Cucurbitaceae family, which comprises 47 species in Africa and 12 in Asia and Australia [7]. It grows in the wild in tropical and subtropical Africa, Asia, America, and the Caribbean and can also be cultivated [8]. Since ancient times, in many countries and regions bitter melon has been used as an herbal medicine for the treatment of diabetes, and it still plays an important role in the prevention and remedy of diabetes in many developing countries [9,10,11]. The extract from its fruits, vines, leaves, and even its roots have been used as folk medicine for the remedy of toothache, diarrhea, and furuncle. Moreover, it is also used for the treatment of dysmenorrhea, eczema, gout, jaundice, leprosy, piles, pneumonia, psoriasis, rheumatism, and scabies [12]. Up to now, numerous studies regarding the biological activities of *M. charantia* have been done, such as its hypoglycemic, anti-bacterial, anti-viral, anti-tumor, immunomodulation, anti-oxidant, anti-diabetes, anthelmintic, antimutagenic, antilipolytic, antifertility, hepatoprotective, and anti-ulcerogenic properties [13]. These properties are due to the complex chemical composition of *M. charantia,* which includes tannins, terpenoids, carbohydrates, resins, saponins, flavonoids, sterols, phylobatamins, anthraquinones, glycosides, amino acids, fatty acids, and phenolic compounds [7].

Wild bitter melon (*Momordica charantia* L. var. Abbreviata Ser.) is a wild edible variety of *M. charantia*, often used in folk medicine. It is normally smaller than cultivated bitter melon, and the genetic similarity between it and the cultivated variety is 80–98% [14]. Recent studies have reported certain biological activities of wild bitter melon, which have been instrumental in improving metabolic syndrome [15], countering alcoholic fatty liver [16], and reducing inflammation [17]. Further studies relating its biological activities are still limited. In this study, the biological activities of wild bitter melon, including scavenging free radicals, inhibiting α-amylase activity, and reducing the production of a pro-inflammatory mediator, were investigated in vitro.

## 2. Materials and Methods

### 2.1. Materials

Wild bitter melon was collected from Binh Phuoc Province, Vietnam, and the solvent was purchased from Xilong (Guangdong, China). Acarbose and Metformin were purchased from a pharmacy store at district 7, Ho Chi Minh city, Vietnam. All other reagents were purchased from Sigma–Aldrich (St. Louis, MO, USA).

### 2.2. Extraction

Wild bitter melon was air-dried in the shade and ground to a powder using a grinder. The powder was soaked with ethanol under the extract conditions ratio (1/4, *w*/*v*), during 4 h at a temperature of 60 °C. The ethanol extract (ET) was subsequently suspended in distilled water and partitioned successively with organic solvents to yield petroleum ether (PE), chloroform (CF), ethyl acetate (EA), n-butanol (Bu), and a water-soluble residue (H_2_O). The extract was dissolved in dimethyl sulfoxide (DMSO) 10% for further investigation.

### 2.3. 1,1-Diphenyl-2-Picryl-Hydrazyl Assay

DPPH (1,1-diphenyl-2-picryl-hydrazyl) scavenging assay was conducted as described by Vo et al. [18]. Briefly, the mixture of 100 μL of extract (0.1, 0.2, 0.3, 0.4, 0.5, and 0.6 mg/mL) or Vitamin C (20 µg/mL) and 100 μL of DPPH solution was incubated in the dark for 30 min at room temperature. The absorbance of the mixture was then measured at 490 nm using Genova Nano (Jenway, UK). The DPPH scavenging ability of the extract was determined following the formula:

DPPH scavenging activity = [(OD_control_ − OD_sample_)/OD_control_] × 100%, (1)

where OD is the optical density.

### 2.4. 2,2-Azinobis-3-Ethyl Benzothiazoline-6-Sulfonic Acid Assay

ABTS (2,2-azinobis-3-ethyl benzothiazoline-6-sulfonic acid) scavenging assay was performed as described by Vo et al. [18]. The mixture of 900 µL of ABTS^+^ solution and 100 µL of the tested sample at different concentrations (0.1, 0.2, 0.3, 0.4, 0.5, or 0.6 mg/mL) was mixed for 45 s and kept for 15 min. The absorbance value of the mixture was then measured at wavelength of 734 nm. Vitamin C (20 µg/mL) was used as a positive control. The ABTS^+^ scavenging ability of the extract was determined following the formula:

ABTS^+^ scavenging activity = [(OD_control_ − OD_sample_)/OD_control_] × 100%. (2)

### 2.5. DNA Oxidative Assay

The protective activity of the tested sample against DNA oxidation was examined as described by Vo et al. [19]. Briefly, the mixture was prepared by adding the tested sample, FeSO_4_ (200 µM), H_2_O_2_ (500 µM), and the isolated genomic DNA (from human liver cancer cell line, HepG2). The reaction was maintained for 10 min before adding EDTA (10 mM). Vitamin C (20 µg/mL) was used as the positive control, while DMSO 10% was used as the negative control. The DNA visualization was conducted by electrophoresis of agarose gel 1% for 20 min at 100 V before stained with ethidium bromide.

### 2.6. α-Amylase Inhibitory Assay

This assay was based on the starch iodine method as described by Daksha et al. [20]. The mixture was firstly prepared by adding 1 mL of the starch (1%, *w*/*v*), 1 mL of the tested samples or acarbose, or DMSO 10%, 1 mL of α-amylase enzyme (*Bacillus licheniformi*, 1U/mL), and 2 mL of acetate buffer (0.1 M, 7.2 pH). It was then incubated for 1 h at 37 °C before adding iodine-iodide indicator. Ultraviolet-visible light (UV-Vis) spectroscopy was used to measure the absorbance at 565 nm.

Inhibition of α-amylase (%) = [(OD_sample_ − OD_control_)/OD_sample_] × 100%. (3)

### 2.7. Cell Culture

The medium used for cell culture contains dulbecco’s modified eagle medium (DMEM), 10% heat-inactivated fetal bovine serum (FBS), 2 mM of l-glutamine, 10 mM of 4-(2-hydroxyethyl)-1-piperazineethanesulfonic acid (HEPES), 100 U/mL of penicillin G, and 100 mg/mL of streptomycin. The cells were maintained in an incubator containing 5% CO_2_ at 37 °C.

### 2.8. Nitrid Oxide Production Assay

Nitric oxide (NO) production level was quantified as described by Vo et al. [21]. Briefly, RAW 264.7 cells (1 × 10^5^ cells/mL) were plated in 96-well culture plates, and subsequently treated with the tested sample or Ibuprofen (0.1 mg/mL) or DMSO 10% for 12 h prior to stimulation of LPS (1 µg/mL) for 12 h. The supernatant (50 µL) was collected and mixed with Griess reagent (50 µL) for 15 min. The control group was stimulated with LPS without sample treatment. Ultraviolet-Vis spectroscopy was used to measure the absorbance at 540 nm. The standard curve of sodium nitrite was applied to measure nitrite concentration in the cultured supernatant.

### 2.9. Cell Viability Assay

The cytotoxic effect of the tested sample on the cultured cells was examined by 3-(4,5-dimethylthiazol-2yl)-2,5-diphenyl-tetrazolium bromide (MTT) method. Briefly, cells (1 × 10^5^ cells/mL) were treated with the tested sample or Ibuprofen (0.1 mg/mL) or DMSO 10% for 24 h before adding MTT solution (1 mg/mL) for 4 h. Subsequently, the supernatant was removed, and 100 µL of DMSO was added. The absorbance was measured at a wavelength of 540 nm by a microplate reader.

### 2.10. Statistical Analysis

Statistical analysis was performed by using the analysis of variance (ANOVA) test of statistical package for social sciences (SPSS, Chicago, IL, USA). The statistical significance of differences among groups was analyzed using Duncan’s multiple range test. *p* < 0.05 was considered significant.

## 3. Results and Discussion

### 3.1. Free Radical Scavenging Activities of Wild Bitter Melon 

**Figure 1:**
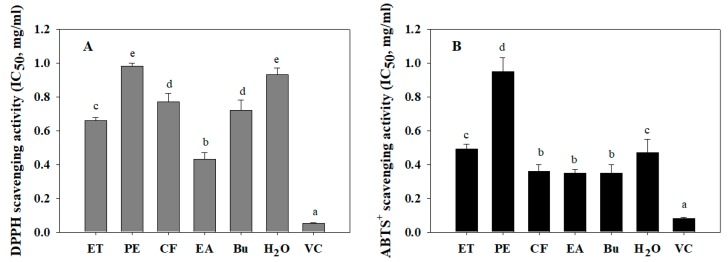
1,1-diphenyl-2-picryl-hydrazyl (DPPH) (**A**) and 2,2-azinobis-3-ethyl benzothiazoline-6-sulfonic acid (ABTS)^+^ (**B**) scavenging activities of wild bitter melon extract and fractions. IC_50_ is the half maximal inhibitory concentration of extract or fractions on DPPH or ABTS^+^ radicals. Each determination was made in three independent experiments, and the data are shown as means ± standard deviation (SD). Different letters a–e indicate significant differences among groups (*p* < 0.05). Ethanol extract (ET), petroleum ether (PE), chloroform (CF), ethyl acetate (EA), n-butanol (Bu), H_2_O fraction, and Vitamin C (VC, 20 µg/mL).

Free radicals are produced by chemical reactions in living organisms, or they are derived from external sources such as exposure to X-rays, foods, cigarette smoking, air pollutants, and industrial chemicals. The increase in the level of free radicals in the human body could cause damage to the body’s tissues and cells, leading to premature aging and various diseases [22,23]. Thus, the consumption of natural products with a high anti-oxidant effect is necessary for the prevention and treatment of diseases caused by free radicals [23]. Numerous methods have been applied in the investigation of anti-oxidative activity, including DPPH and ABTS radical assays. The principle behind these assays concerns the donation of a hydrogen atom or electron of antioxidant agent to neutralize DPPH and ABTS^+^ radicals. The neutralized form will lose color, and the antioxidant ability can be monitored by spectrophotometer. Herein, the anti-oxidant activity of wild bitter melon extract and fractions was determined by measuring the scavenging ability on DPPH and ABTS^+^ radicals, as seen in Figure 1. In Figure 1A, the highest DPPH scavenging activity was observed in EA fraction (half maximal inhibitory concentration (IC_50_) = 0.43 ± 0.04 mg/mL), followed by ET extract (IC_50_ = 0.66 ± 0.017 mg/mL), Bu fraction (IC_50_ = 0.72 ± 0.06 mg/mL), CF fraction (IC_50_ = 0.77 ± 0.05 mg/mL), H_2_O fraction (IC_50_ = 0.93 ± 0.04 mg/mL), and PE fraction (IC_50_ = 0.98 ± 0.021 mg/mL). Meanwhile, CF, EA and Bu fractions exhibited the highest ABTS^+^ scavenging activity with IC_50_ values of 0.36 ± 0.04 mg/mL, 0.35 ± 0.02 mg/mL, and 0.35 ± 0.05 mg/mL, respectively, followed by H_2_O fraction (IC_50_ = 0.47 ± 0.08 mg/mL), ET extract (IC_50_ = 0.49 ± 0.03 mg/mL), and PE fraction (IC_50_ = 0.95 ± 0.08 mg/mL), as can be seen in Figure 1B. The radical scavenging activity of vitamin C was four to eight times higher than that of EA fraction. Moreover, DMSO 10%, which was used to dissolve the extract, had no antioxidant activity (Figure S1). Generally, solvents with different polarities were applied to isolate antioxidants with different polarities. In this study, the solvents with moderate polarity, such as ethyl acetate and n-butanol, were found to be effective in extracting potential DPPH and ABTS^+^ radical scavenging compounds. Similarly, Rezaeizadeh and colleagues also determined DPPH scavenging activity of methanol and chloroform extract of *M. charantia* with IC_50_ values of 0.31 and 0.58 mg/mL, respectively [24]. Aljohi and colleagues showed that the aqueous extracts of *M. charantia* flesh and *M. charantia* pulp were not effective in scavenging DPPH radical. These extracts brought about 50% DPPH scavenging activity at the concentration of 15 mg/mL) [25].

### 3.2. Protective Effect of Wild Bitter Melon against H_2_O_2_-Induced DNA Damage

**Figure 2:**
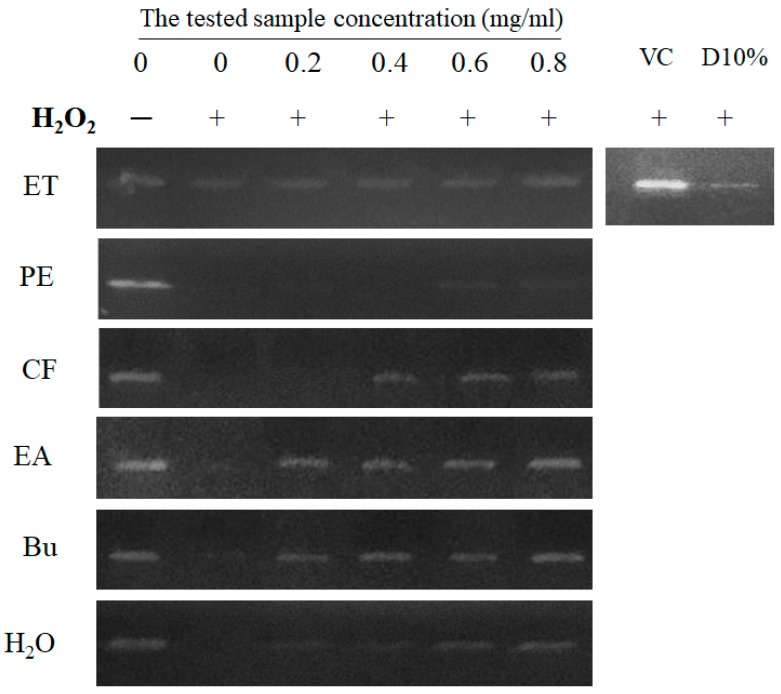
Protective effects of wild bitter melon extract and fractions against H_2_O_2_-induced DNA damage. Dimethyl sulfoxide (DMSO) 10% (D10%) was used as negative control, while vitamin C (VC) was used as positive control. “+” indicates the presence of H_2_O_2_, while “−” indicates the absence of H_2_O_2_. The control was exposed by H_2_O_2_ without the tested sample, while the blank was absent both H_2_O_2_ and the tested sample. Ethanol extract (ET), petroleum ether (PE), chloroform (CF), ethyl acetate (EA), n-butanol (Bu), and H_2_O fraction.

Oxygen-containing free radicals such as hydroxyl radical, superoxide anion radical, hydrogen peroxide, oxygen singlet, and superoxide anion radical are highly reactive species which cause DNA damage, the progress of atherosclerosis, inflammatory condition, certain cancers, and aging [26]. Therefore, anti-oxidant agents play an important role in protecting DNA from oxidative damage via donating an electron or hydrogen atom to neutralize the rampaging free radicals. In this study, the protective effect of extract and fractions of wild bitter melon against H_2_O_2_-induced DNA damage was investigated. It was observed that the radicals generated by Fenton reaction broke the DNA to small fragments. Thus, the DNA band did not appear in the control group clearly (Figure 2). Conversely, the DNA band in the blank group was visible as a sharp and bright band compared to the control. As the result, free radical scavenging agents are indicated by the protective capacity against DNA damage induced by radicals. Interestingly, EA and Bu fractions exhibited the significantly protective effect against H_2_O_2_-induced DNA damage in concentration-dependent manner. Especially, a DNA band at 0.8 mg/mL of EA or Bu treatment is visible as a sharp band near the blank. It indicates the protective effect of EA and Bu fractions on DNA molecules from radical damage. The ET, CF, and H_2_O were observed to possess the moderate protective effect, while PE and DMSO 10% were not effective in the protection against H_2_O_2_-induced DNA damage. Vitamin C, a positive control, exhibited to be effective in protecting DNA from radical damage.

### 3.3. α-Amylase Inhibitory Activity of Wild Bitter Melon

**Figure 3:**
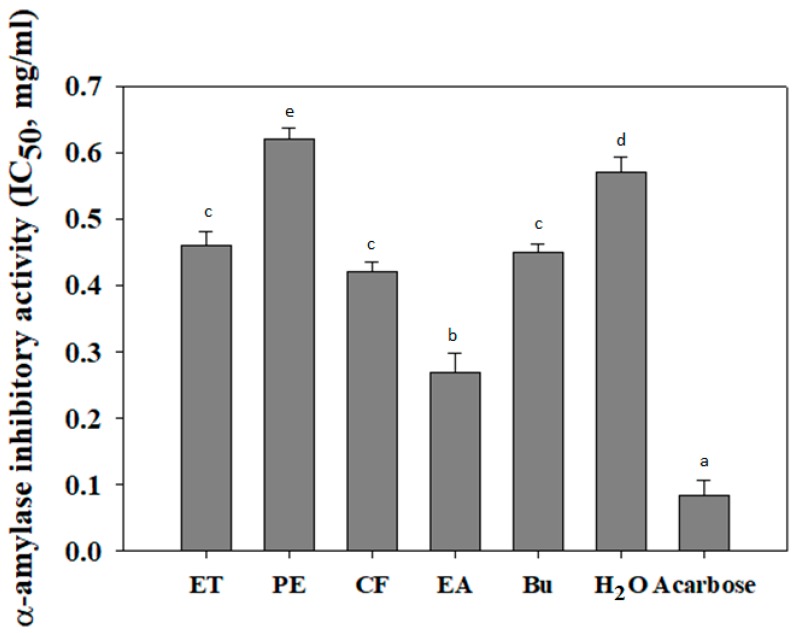
The inhibitory effect of wild bitter melon extract and fractions on α-amylase activity. IC_50_ is the half maximal inhibitory concentration of extract or fractions on α-amylase activity. Acarbose was used as the positive control. Each determination was made in three independent experiments, and the data were shown as means ± SD. Different letters a–e indicate significant differences among groups (*p* < 0.05). Ethanol extract (ET), petroleum ether (PE), chloroform (CF), ethyl acetate (EA), n-butanol (Bu), and H_2_O fraction.

Postprandial hyperglycemia is known to relate to high blood glucose level after meal consumption [27]. Notably, α-amylase enzyme plays an important role in carbohydrate digestion and postprandial glucose increase in diabetic patients. Thus, the inhibition of α-amylase activity can reduce postprandial hyperglycemia and prevent the risk of diabetes [28,29]. Recently, wild bitter melon has been emerged as an alternative medicine for prevention and/or treatment of type 2 diabetes. However, its inhibitory activity on α-amylase activity was not obviously reported. In this study, wild bitter melon extract and its fractions were determined to suppress α-amylase activity. As shown in Figure 3, the lowest IC_50_ values were identified by EA fraction (0.27 ± 0.029 mg/mL), followed by CF, Bu, ET, H_2_O, and PE (0.042 ± 0.015, 0.045 ± 0.012, 0.46 ± 0.021, 0.57 ± 0.023 mg/mL, and 0.62 ± 0.018, respectively). It indicates that EA fraction possesses the highest α-amylase inhibitory activity as compared to others. Meanwhile, the α-amylase inhibitory activity of acarbose was higher than that of EA fraction. In another study, protein extract from *M. charantia var. charantia* and *M. charantia var. muricata* were shown to inhibit α-amylase activity with IC_50_ values of 0.267 ± 0.024 mg/mL and 0.261 ± 0.019 mg/mL, respectively [30]. Moreover, polysaccharide extracted from *M. charantia* also inhibited α-amylase activity with an IC_50_ value of 6.69 mg/mL [31]. As a result, the inhibition of EA fraction from wild bitter melon on α-amylase activity was observed to be similar with that of *M. charantia* protein extract and to be higher than that of *M. charantia* polysaccharide.

### 3.4. The Inhibitory Activity of Wild Bitter Melon on Nitric Oxide Production

**Figure 4:**
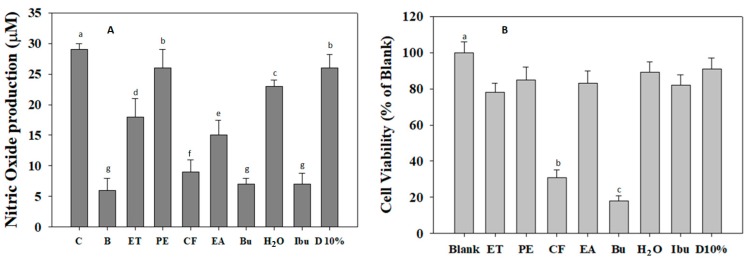
(**A**) The suppressive effect of wild bitter melon extract and fractions on nitric oxide (NO) production from LPS-stimulated RAW 264.7 cells. NO level was measured by the Griess reaction. Ibuprofen (Ibu) was used as positive control. DMSO 10% (D10%) was used as negative control. Control (C) was stimulated with LPS without the tested sample treatment, while blank (B) was free LPS and the tested sample. (**B**) The effect of wild bitter melon extract and fractions on RAW 264.7 cell viability. Blank is free the tested sample. DMSO 10% (D10%) was used as negative control. Cell viability was assessed by MTT method, and the results were expressed as percentage of surviving cells over blank cells. Each determination was made in three independent experiments, and the data were shown as means ± SD. Different letters a–g indicate significant difference among groups (*p* < 0.05). Ethanol extract (ET), petroleum ether (PE), chloroform (CF), ethyl acetate (EA), n-butanol (Bu), and H_2_O fraction.

Evidently, NO is a pro-inflammatory mediator which plays a key role in the pathogenesis of inflammation [32]. Therefore, the inhibition of NO production represents an important therapeutic advance in the control of inflammatory diseases. In this sense, the RAW 264.7 cells were pre-treated with wild bitter melon extract and fractions before the stimulation of lipopolysaccharide (LPS), and the level of NO production was then measured in supernatant cultures. Obviously, LPS triggered the production of NO from RAW 264.7 cells up to 29 µM. Conversely, NO production levels were decreased by the extract and fractions treatment (Figure 4A). Especially, CF and Bu fractions significantly reduced the NO production levels to 9 ± 2 µM and 7 ± 2 µM, respectively. The moderate inhibition was observed by ET extract and EA fraction (18 ± 2 µM and 15 ± 2.5 µM, respectively), while PE and H_2_O fractions exhibited slight inhibition on NO production (26 ± 3 µM and 23 ±1 µM, respectively). The inhibitory activity of Ibuprofen on NO production was similar with that of CF and Bu fractions and higher than that of EA fraction. Meanwhile, DMSO 10% was not effective in α-amylase inhibition. It indicates that the inhibition of NO production by wild bitter melon is not due to DMSO 10%. To exclude the possibility that the inhibition of NO production was due to cytotoxic effect, MTT assay was conducted on RAW 264.7 cells pre-treated with wild bitter melon extract and fractions for 24 h. As shown in Figure 4B, CF and Bu fractions markedly caused cytotoxic effect on RAW 264.7 cells, while ET extract, PE, EA, and H_2_O fractions, Ibuprofen, and DMSO 10% exhibited no significant cytotoxicity. Accordingly, the inhibitory effect of CF and Bu fractions on NO production is suggested due to cytotoxicity effect. As a result, ET extract and EA fraction were considered to be potential agents for the amelioration of inflammatory reaction.

Currently, wild bitter melon is commonly used as food or medicine in several forms by being eaten as fruit, drunk as juice, or boiled in water as a decoction. Alternatively, wild bitter melon extract can be consumed as an herbal supplement, such as Glycostat (Glykon Technologies, Las Vegas, NV, USA). It has been used to support cardiovascular and metabolic functions as well as to control blood pressure and blood glucose into the normal range. So far, about 228 different medicinal compounds have been isolated from several parts of bitter melon including glycosides, saponins, alkaloids, triterpenes, proteins, steroids, carotenoids, monoterpenes, carbohydrates, benzanoids, and sesquiterpenes [33]. Especially, its stem and leaves were found to contain various biologically active chemicals such as Cucurbitane-type triterpenoids [34], saponin [35], Kuguacins F–S [36], vicine [37], and charantin [38]. These compounds may be responsible for the free radical scavenging activity, α-amylase inhibitory activity, and NO production suppression from macrophage cells in the present study. However, further studies regarding the purification of active compounds and the evaluation of their biological activities is necessary.

## 4. Conclusions

In conclusion, the present study indicates different potential biological activities of wild bitter melon. It evidences that EA fraction possessed high anti-oxidant activity via scavenging DPPH and ABTS^+^ radicals and reducing H_2_O_2_-induced DNA damage. Moreover, EA fraction was effective in the inhibition of α-amylase activity and suppression of pro-inflammatory mediator production in vitro. Therefore, ethyl acetate is suggested as a proper solvent for the extraction of bioactive components with high biological activities from wild bitter melon. Moreover, the active EA fraction could be considered as a promising ingredient of functional food for health promotion.

## Supplementary Materials

The following are available online at https://www.mdpi.com/2218-273X/9/6/211/s1, Figure S1: DPPH and ABTS^+^ scavenging effect of DMSO 10%.
