# Supplementary material for: Investigation of Biological Activities of Wild Bitter Melon (Momordica charantia Linn. Var. Abbreviata Ser.)
Source: Biomolecules. 2019 May 30;9(6):211. doi: 10.3390/biom9060211 (PMC6627102; doi:10.3390/biom9060211)
Supplement: Supplementary file 1 [file biomolecules-09-00211-s001.pdf]

Supplementary Figure

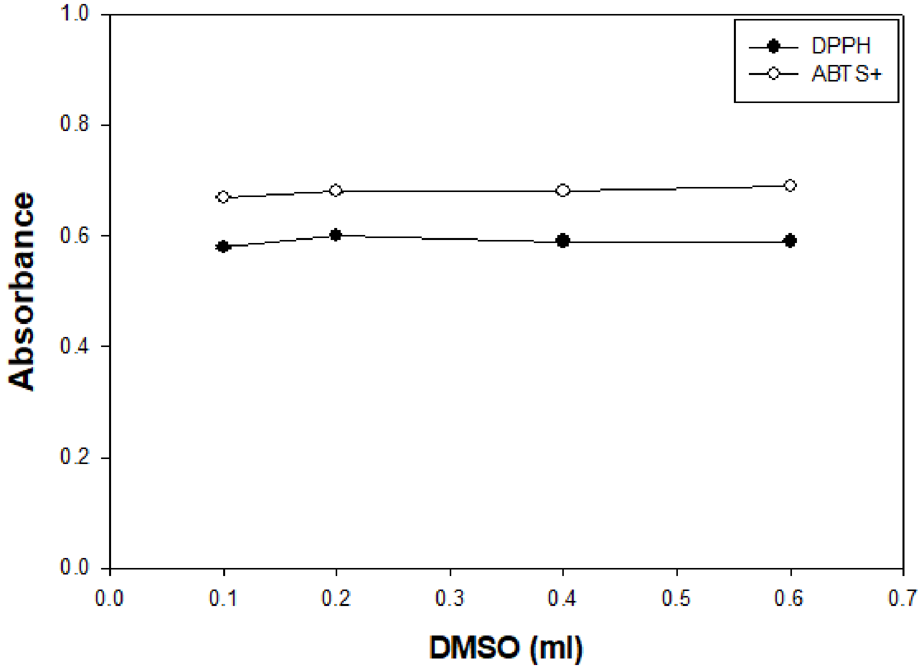

Figure S1. DPPH and ABTS+ scavenging effect of DMSO 10%. DMSO 10% was mixed with DPPH solution and subsequently incubated in the dark at room temperature for 30 min. The absorbance of the mixture was then measured at 490 nm. Besides, DMSO 10% was mixed with ABTS+ solution and subsequently incubated in the dark at room temperature for 15 min. Measurement was taken at 734 nm. DMSO: Dimethyl sulfoxide.
